# No Interaction Effect between Interleukin-6 Polymorphisms and Acid Ash Diet with Bone Resorption Marker in Postmenopausal Women

**DOI:** 10.3390/ijerph18020827

**Published:** 2021-01-19

**Authors:** Sook Yee Lim, Yoke Mun Chan, Vasudevan Ramachandran, Zalilah Mohd Shariff, Yit Siew Chin, Manohar Arumugam

**Affiliations:** 1 Department of Nutrition and Dietetics, Faculty of Medicine and Health Sciences, University Putra Malaysia (UPM), UPM Serdang, Seri Kembangan 43400, Selangor, Malaysia; l.sookyee@yahoo.com (S.Y.L.); zalilahms@upm.edu.my (Z.M.S.); chinys@upm.edu.my (Y.S.C.); 2 Research Center of Excellence Nutrition and Non-Communicable Diseases, Faculty of Medicine and Health Sciences, University Putra Malaysia (UPM), UPM Serdang, Seri Kembangan 43400, Selangor, Malaysia; 3 Malaysian Research Institute on Ageing, University Putra Malaysia, UPM Serdang, Seri Kembangan 43400, Selangor, Malaysia; 4 Department of Orthopedics, Faculty of Medicine and Health Sciences, University Putra Malaysia (UPM), UPM Serdang, Seri Kembangan 43400, Selangor, Malaysia; a_manohar@upm.edu.my

**Keywords:** dietary acid load, rs 1800795, rs 1800796, bone loss, Asian Chinese, CTX1

## Abstract

Background: Evidence is growing that a high-acid diet might accelerate the rate of bone loss, and gene polymorphisms such as Interleukin 6 (*IL6*) -174G/C and -572G/C are related to bone deterioration. However, no study of the interaction between diet and *IL6* polymorphisms has been conducted among Asians. Thus, the objective of this study was to determine whether IL6 gene polymorphisms modified the association between dietary acidity and the rate of bone resorption. Methods: This cross-sectional study recruited 203 postmenopausal women (age ranged from 51 to 85 years old) in community settings. The dietary intakes of the participants were assessed using a validated interviewer-administered semi-quantitative food frequency questionnaire (FFQ), while dietary acid load (DAL) was estimated using net endogenous acid production (NEAP). Agena^®^ MassARRAY genotyping analysis and serum collagen type 1 cross-linked C-telopeptide (CTX1) were used to identify the *IL6* genotype and as a bone resorption marker, respectively. The interactions between diet and single-nucleotide polymorphisms (SNPs) were assessed using linear regressions. Results: A total of 203 healthy postmenopausal women aged between 51 and 85 years participated in this study. The mean BMI of the participants was 24.3 kg/m^2^. In *IL6* -174 G/C, all the participants carried the GG genotype, while the C allele was absent. Approximately 40% of the participants had a high dietary acid load. Dietary acid load (B = 0.15, *p* = 0.031) and the *IL6* -572 CC genotype group (B = 0.14, *p* = 0.044) were positively associated with a higher bone resorption. However, there was no moderating effect of the *IL6* genetic polymorphism on the relationship between and acid ash diet and bone resorption markers among the postmenopausal women (*p* = 0.79). Conclusion: High consumption of an acid ash diet and the *IL6* -572 C allele seem to attribute to high bone resorption among postmenopausal women. However, our finding does not support the interaction effect of dietary acidity and *IL6* (-174G/C and -572G/C) polymorphisms on the rate of bone resorption. Taken together, these results have given scientific research other candidate genes to focus on which may interact with DAL on bone resorption, to enhance planning for preventing or delaying the onset of osteoporosis among postmenopausal women.

## 1. Introduction

A bourgeoning body of research has focused on osteoporotic fractures in light of their great impact on public health [1]. Osteoporotic-related fractures may lead to disability, comorbidity (e.g., functional intestinal disorder [2], heart failure, acute renal failure, thromboembolic and cirrhosis complications) [3], increased medical costs, decreased quality of life, and mortality [4]. In Asia, the incidence of osteoporotic fractures is estimated to rapidly increase and it is estimated that 50% of all osteoporotic hip fractures worldwide will occur in Asia by the year 2050 [5]. In a multiracial country such as Malaysia with three main ethnicities—namely, Malays, Chinese, and Indians—Malaysian Chinese females aged 50 years and above are more susceptible to osteoporotic fractures [6]. Nonetheless, the interplay between dietary factors and genetics factors, which is expected to play an important role in bones, has received little attention.

Despite being the gold standard for the quantitative assessment of bone and an important predictor of fracture risk [7], bone mineral density (BMD) measurement using dual-energy X-ray absorptiometry (DEXA) is not able to assess bone quality [8] and requires a long duration to detect BMD changes. On the other hand, biochemical bone turnover markers (BTMs), which are able to evaluate the dynamics of bone remodeling in terms of the rate of bone formation and resorption, are widely used to offer clinical information for predicting fracture risk and monitoring the efficacy of anti-osteoporosis therapy [9,10]. Among BTMs, serum-procollagen type I N-propeptide (PINP) and serum C-terminal telopeptide of type I collagen (CTX1) are the most promising reference markers to detect bone formation and bone resorption for use in clinical medicine, respectively [11,12]. 

Attributable to the complex predominance of osteoclastic activity, genetic polymorphism factors contributed up to 80% in determining BMD [13]. Interleukin 6 (*IL6*) single-nucleotide polymorphism (SNP) is one of the susceptible genes that is commonly used to detect bone deterioration [14]. *IL6* is a key moderator of inflammation which has been found to play an important role in the pathogenesis of atherosclerosis, while *IL6* gene -174G/C (rs 1800795) and -572G/C (rs 1800796) polymorphisms were related to bone metabolism and BMD among postmenopausal women [15]. Several studies identified *IL6* -572G/C polymorphism as being common in the Asian population [16,17,18]. Among all, Pan et al. [19] reported that approximately 60% of the Han Chinese population carry the CC genotype, while 40% carry GG + CG genotypes. On the other hand, nearly all of the *IL6* -174 G/C polymorphism studies were among Europeans, with a scarcity of data from Asians. Sun et al. [20] showed that nearly one third of their subjects had either CC or GC genotypes in their coronary artery disease study in China, indicating that the Chinese population may have the *IL6* -174 G/C polymorphism too.

Dietary factors are other independent factors that may affect body acidity and the rate of bone loss. Generally, a highly acidic diet is recognized as a diet that is high in animal proteins and may generate endogenous acids. Although body acidity is generally tightly regulated, the habitual intake of a high-acid ash diet without adequate compensation by alkaline ions such as potassium and magnesium from fruit and vegetables may lead to a lower blood pH [21], which may affect the acid–base homeostasis in the body [22]. Despite the acid base balance theory hypothesized, the habitual consumption of a high acid-load diet and an inadequate consumption of potassium and bicarbonate-rich (base-load diet) is associated with an increase in urinary calcium, magnesium loss, and consequently a higher risk of osteoporosis. The evidence of the influence of DAL on bone health has been inconsistent [23,24,25,26,27]. The contradictory findings could be related to a lack of consideration of the links between DAL and genetic factors.

On the other hand, while there is growing evidence that *IL6* (-174G/C and -572G/C) genetic polymorphisms with the presence of C alleles is associated with a higher risk of bone resorption, the potential association between dietary acidity and bone health remains inconclusive. Therefore, we hypothesized that the complex interaction between *IL6* (-174G/C and -572G/C) genetic polymorphisms and dietary acidity may be implicated in lower bone mass. To date, this is the first study to examine how genetic factors may modify the association between diet and bone resorption among postmenopausal women. We aimed to explore whether *IL6* SNPs modulate the effect of dietary acidity on bone resorption among postmenopausal women.

## 2. Materials and Methods 

### 2.1. Study Population

**Figure 1:**
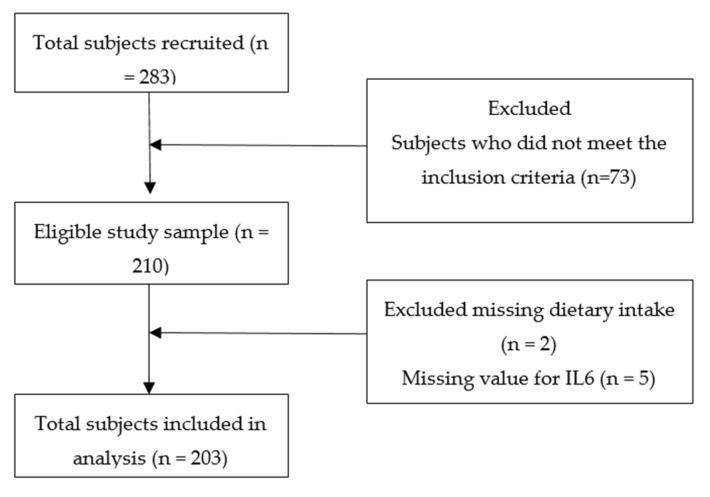
Flow diagram of the study.

A total of 203 postmenopausal women with ages ranging from 51 to 85 years old was randomly recruited from National Council of Senior Citizens Organizations Malaysia (NACSCOM). This was an analytical cross-sectional study conducted on a representative population (Chinese women that have been postmenopausal for five years or more, not on any medications that may affect bone health within the past one year). The exclusion criteria were the presence of any systemic diseases (e.g., cardiovascular diseases, cancer, stroke, impaired liver and renal function) and on medication (e.g., hormone replacement therapy, aluminum-containing antacids, anticonvulsants, aromatase inhibitors, immunosuppressant, glucocorticoids, proton pump inhibitors, selective serotonin reuptake inhibitors, and thiazolidinediones) that could affect bone health within the past one year. Figure 1 shows the flow diagram of the study. The sample size was determined using the Gpower software using a number of predictors set at nine (age, height, serum of 25(OH)D, education level, fasting blood glucose, waist circumference, NEAP, IL6, and bone resorption); the effect size f^2^ was 0.15, which gave a study power of 95%. The written informed consent of participants were obtained prior to study enrollment. The study protocol was approved by the Ethics Committee for Research Involving Human Subjects (project reference number FPSK (FR16) P019).

### 2.2. Measurements 

The sociodemographic factors of participants were obtained using a pre-tested structured questionnaire. Anthropometric measurements including height, weight, and waist circumference were ascertained using standardized techniques [28,29]. A universal surrogate measure of body weight status, body mass index (BMI), was computed as the ratio of weight (kg) to height^2^ (m^2^), and was classified according to the WHO (2000) [30]. The percentage of body fat of the participants was measured using the body fat monitor HBF-306 (Omron Matsusaka Co. Ltd., Matsusaka, Japan) with an accuracy of up to 0.1%. Blood pressure was assessed using a Digital Automatic BP monitor (OMRON HEM-907, Omron, Kyoto, Japan), while the physical activity level was obtained using the validated Global Physical Activity Questionnaire (GPAQ) [31]. The adequacy of physical activity among the participants was ascertained according to the recommendation by the WHO whereby an individual should perform more than 600 Metabolic Equivalent (MET) minutes of total physical activity per week, with MET (Metabolic Equivalent) defined as “the ratio of a person’s working metabolic rate relative to the resting metabolic rate” in the GPAQ analysis guide [32].

### 2.3. Dietary Assessment

The habitual food intake of participants over the past one month was assessed using a validated 165-item semi-quantitative food frequency questionnaire (FFQ) [33], which was adapted from the Malaysian Adult Nutrition Survey (MANS) [34]. The food items were foods that are commonly consumed by the Malaysia population and were categorized according to 14 food groups: cereals and cereal products, fast food, meat and meat products, fish and seafood, eggs, legumes and legume products, milk and milk products, vegetables, fruits, drinks, alcoholic drinks, confectionaries, bread spread, and seasonings. The frequency intake of each food item on a daily, weekly, or monthly basis was converted to daily intakes; portion sizes were converted to grams, based on the household measurement listed in the Album Makanan Malaysia [35]. Nutrients were computed using the Nutritionist Pro™ Diet Analysis (Axxya Systems, Stafford, TX, USA) software, with the Nutrient Composition of Malaysia Foods [36] and Singapore Food Composition Database [8] as the primary databases. Several studies showed that the FFQ provides a good validity and reliability to assess the long-term nutrient intake for the estimation of dietary acidity [37,38,39]. Potential renal acid load (PRAL) and daily net endogenous acid production (NEAP) are the two common algorithm calculations used to estimate the acid load from dietary intake. Both NEAP and PRAL are highly correlated with acid load measured from 24 h urine samples in healthy men and women [40,41], making them suitable as surrogate measures for dietary acid load. In this study, dietary acidity was determined using the following equation: NEAP (mEq/d) = [54.5 × protein intake (g/d)/potassium intake (mEq/d)]−10.2 [40]. 

### 2.4. Biochemical Measurements

A total of 10 mL of fasting blood was collected between 9:00 and 10:30 h by certified phlebotomists and was then sent to a certified commercial laboratory for biochemical analysis. The fasting blood glucose was estimated by the Hexokinase method performed by the Olympus AU analyzer. The serum levels of 25(OH) vitamin D were determined using the Siemens ADVIA Centaur Vitamin D Total assay (Siemens, Tarrytown, NY, USA), with the analytical measuring range of 4.2 to 150 ng/mL (10.5 to 375 nmol/L). This assay has been standardized to the University of Ghent ID-LC/MS/MS reference measurement procedure and has achieved the Centers for Disease Control Vitamin D Standardization Certification program [35]. The vitamin D statuses of participants were classified into three subgroups according to the recommendation by the Institute of Medicine (2011) [36] as deficient (<30 nmol/L), inadequate (30–50 nmol/L), or adequate (>50 nmol/L), respectively. On the other hand, the serum CTX1 was assessed by a fully automated analyzer (Elecsys 2010, Roche Diagnostics, GmbH, Mannheim, Germany), which can reduce the variability and is suitable for routine use in clinical chemistry [37].

### 2.5. Genetic Analysis

Genomic DNA was extracted from non-coagulated whole blood samples (EDTA tube, Becton Dickinson, NJ, USA) using a commercially available DNA extraction kit (QIAamp DNA Blood Mini Kit Qiagen, Hilden, Germany) according to the manufacturer’s protocol. The quality of the extracted DNA was evaluated by means of electrophoresis, and the concentration of the extracted DNA was estimated using the spectrophotometer. For the SNP control, built-in controls were used to easily quantify amplifiable copies of DNA. The panel includes 5 DNA copy number control assays (albumin) to quantify from as little as 500 to 18,000 amplifiable copies (~1 ng to 60 ng) of DNA. Each genotyping was performed with the Agena^®^ MassARRAY platform. After the SNP detection process, the Typer Analyzer was used to analyze the output data from the Agena^®^ MassARRAY platform.

### 2.6. Statistical Analysis

The Statistical Package for Social Sciences for Windows version 22.0 (SPSS, Chicago, IL, USA) was used to perform the statistical analysis. The data normality was checked by a normal P-P plot of the standardized residuals. First, the descriptive data were expressed as mean ± SD or frequency and percentage. In light of the absence of established cut offs for NEAP and CTX1, the mean values of the participants were arbitrarily used to identify the overall dietary acid load and rate of bone resorption of the participants. Second, bivariate analyses of Pearson’s correlation were used to determine the relationships between CTX1 with NEAP, sociodemographic background (age and education level), anthropometry parameters (waist circumference and height), and biochemical indices (serum vitamin D and fasting blood glucose). Next, the Hardy–Weinberg equilibrium for genotypic distribution was examined by the Hardy–Weinberg equilibrium exact test. For the IL6 gene -572 G/C polymorphism, with a small number of GG homozygous (5 participants), GG and CG genotypes were collapsed into one single group. Comparisons of the study variables by the IL6 gene -572 G/C polymorphism were examined by the t test (continuous variables) or chi-square test (two categorical groups).

**Figure 2:**
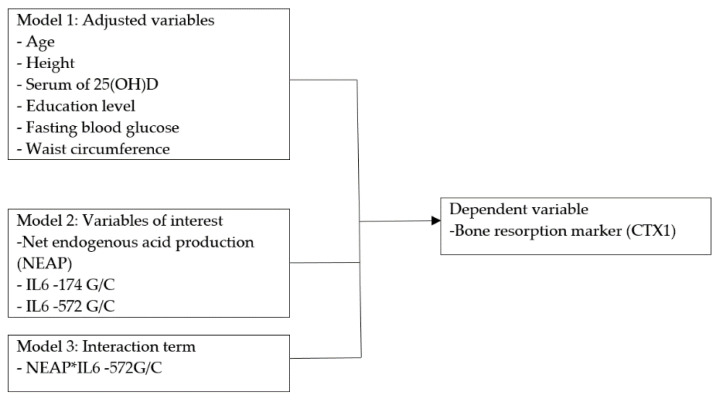
Directed acyclic graph diagram of the hierarchical regression model.

A hierarchical multiple linear regression model was used to determine the interaction effect between an acid ash diet and SNPs, and also to determine the contribution of adjusted variables and variables of interest (acid ash diet and IL6 SNPs) to bone resorption. Hierarchical regression is more flexible, as it allows the researcher to specify the order of entry of the independent variables in the regression equation [42]. Among the theoretic reasons, well-known risk factors such as age, height, serum of 25(OH)D, education level, fasting blood glucose, and waist circumference are the strongest predictors of the dependent variable (CTX1) [43]. Thus, they were dictated as a set of adjusted variables and were accorded priority of entry, and their total amount of variance was evaluated. Then, the interest of variables (NEAP and IL6 SNPs) were entered and evaluated in terms of what they added to the explanation power (total amount of variance). Lastly, the interaction effect was added and evaluated in terms of what it adds to the explanation of the dependent variable. Prior to analysis, the quality of the data was checked and an interaction term was added to the linear regression model for testing the SNP–diet interactions on bone resorption. The assumption of linearity, homoscedasticity, independence of error terms, normality of error distribution, and the absence of multicollinearity was made before hierarchical regression was performed. Figure 2 demonstrates the directed acyclic graph diagram of the study. 

## 3. Results

### 3.1. Characteristics of Participants

As shown in Table 1, the mean age and duration of menopause of participants was 67 ± 7 years and 16 ± 8 years, respectively. Majority of the participants were married and had lower secondary education. The mean BMI of the participants was 24.3 kg/m^2^, with the majority of them normal or overweight, while approximately 5% were underweight. Less than two thirds of the participants met the recommended duration of physical activity. The mean score of NEAP was 72.8 ± 28.7, with approximately 45% having a high dietary acid load. The prevalence of vitamin D deficiency and inadequacy was extremely high (more than 80%). The mean serum CTX1 of the participants was 45% ± 0.2 ng/mL, and approximately 43% had an elevated rate of bone resorption. The IL6 -174G/C polymorphism was absent in this study population, with all the participants carrying the GG wild type. As the CC and CG genotypes were absent, the comparison and linear regression analysis were therefore not pursued for this SNP. The genotype frequencies for rs 1,800,796 were 2.5%, 41.4%, and 56.1% for GG, CG, and CC, respectively.

### 3.2. Correlations between Variables and CTX1

There was a significant negative correlation between CTX1 and age (*r* = −0.19, *p* = 0.007), while the NEAP, height, serum 25(OH) vitamin D, educational level, fasting blood glucose, and waist circumference of the participants were not associated with the CTX1 (Table 2).

### 3.3. Demographic, Anthropometrics, Lifestyle Factors, and Biochemical Analysis of Participants According to IL6 -572G/C Genotypes

There were no significant genotype-associated differences observed for any of the sociodemographic and anthropometric characteristics, lifestyle factors (physical activity and dietary acidity), biochemical measures (fasting blood glucose, serum 25(OH) vitamin D), and CTX1 (Table 3).

### 3.4. Interaction of IL6 -572G/C with NEAP in Relation to CTX1

The hierarchical regression results to determine the direct and interaction effects between the selected variables and the CTX1 are summarized in Table 4, with model 1 (control variables) accounting for 6.6% of the variance (R^2^ = 0.066). In model 2, the NEAP score (B = 0.15, *p* = 0.031) and *IL6* CC genotype group (B = 0.14, *p* = 0.044) showed positive associations with the CTX1 rate. The inclusion of the NEAP and *IL6* -572G/C increased the explained variance in CTX1 by 3.8%. However, inclusion of the interaction term (NEAP*IL6) in model 3 did not reflect any changes in R Square, and hence did not support the interaction term (NEAP*IL6) associated with CTX1 (R square = 0.000, *p* = 0.79) (Table 4).

## 4. Discussion

The present study was the first study on dietary acidity among Malaysians. The higher NEAP score as compared to studies in Hong Kong among an older Chinese population [44] and Caucasians [39,45,46,47] may be attributed to the nutrition transition of the dietary pattern among Malaysians. This finding corroborates the ideas of Soon and Tee [48], who suggested that dietary patterns in Southeast Asian nations have changed from traditional dietary patterns that are high in fresh, cooked, or pickled fruits and vegetables to Western dietary patterns that are generally high in animal products, wheat, sugars, fats, and salts. Several polymorphism studies have tried to establish the association between polymorphisms of certain cytokines with bone health. *IL6* is a cytokine that involved inflammation and infection responses and was reported to play a role in the pathogenesis of osteoporosis [17,49]. In this study, all the participants carried the *IL6* -174 G/C GG genotype. Our findings are consistent with other Asian studies [50,51,52,53,54,55]. For example, a study in South Korea reported that the CC genotype was absent among adolescent idiopathic scoliosis patients and healthy individuals, with only one participant carrying CG genotype [50]. Similar findings were reported among the Chinese [51,52] and Japanese population [54]. Our result was also supported by a local study conducted among the three major ethnicities (Malay, Chinese, and Indian), which showed that only Indians carried 4% of the CC genotype, while no CC genotype was found among Malay and Chinese people [55]. Therefore, we concluded that the *IL6* polymorphism at −174 is rare and unlikely to contribute significantly to disease susceptibility in the Malaysian Chinese population.

We did not find any significant association between -572G/C genotypes and sociodemographics, anthropometrics, lifestyle factors, or biochemical measures. The results are in line with a Caucasian study conducted in Brazil, indicating that the -572G/C genetic polymorphism was not associated with the fasting blood glucose [56]. Moreover, previous studies [57,58,59] showed that there was no significant association between *IL6* -572G/C with age, BMI, and serum 25(OH)D among Japanese postmenopausal women.

The present study shows that a higher acid ash diet as determined by NEAP score may accentuate the bone resorption marker. Prior studies have suggested a few mechanisms to interpret the influence of diet-induced acidosis on bone metabolisms [40,60,61]. Frick and Bushinsky [61] suggested that a slight decrease in the metabolic pH will result in the depletion of bone calcium by increases in urine calcium without increased calcium absorption in the intestine. It was estimated that the quantity of excess calcium excreted in the urine which associated with the acid ash diet over time could be as high as 24g per year or 480 g over 20 years, equivalent to almost half of an adult’s skeletal mass of calcium [62]. The habitual consumption of a high-acidity diet may lead to a consistent stimulation of osteoclast activity and subsequently osteoporosis. New et al. [63] demonstrated that high NEAP scores were associated with a lower bone mass of the femur, hip, and spine among 1056 women. Recently, Shariati-Bafghi et al. [64] showed that PRAL and NEAP scores were inversely correlated with bone mass, and such a relationship was independent of calcium intake. Another Korean study also reported that a high potassium intake was positively associated with a higher bone mineral density in the femur, lumbar spine, and hip, even with the subjects with a lower calcium intake [65]. Despite the positive evidence described, a systematic review and meta-analysis conducted by Fenton et al. [66] concluded that there was no causal association between an acidic diet and the risk of osteoporosis among adults. The inconclusive results may be influenced by the individual genetic makeup. However, since a long-term acidic diet may influence the osteoclast activity, it is believed that the excessive acid gains from a habitual diet and release into the bloodstream may have a significant impact on bone health. More studies are needed on this aspect.

In this study, the *IL6* -174G/C and -572G/C SNPs were considered as candidate genes for bone resorption because of their potential involvement in regulating the proliferation, differentiation, and maturation of osteoblasts in bone turnover pathways [67]. Postmenopausal women are susceptible to higher bone resorption due to the decreased estrogen level caused by oophorectomy or natural menopause [68]. Estrogen plays a role in the bone protection effect inhibition of osteoclast activity, and reducing the estrogen level that may trigger the expression of *IL6* and accelerate the bone resorption process. The findings of this study indicated that the participants with the CC genotype have a significant positive relationship with bone resorption. To the best of our knowledge, there is no other study that investigates the association between *IL6* –572G/C SNP and bone resorption, despite earlier studies by Lee et al. [50] and Chung et al. [69] reporting that C carrier was associated with increased BMD. As BMD reflects the density of bones but is not a marker of bone resorption, a direct comparison with the above two studies cannot be made. More studies are warranted to delineate such association in future studies.

In this study, we provide the first *IL6* genetic polymorphism and diet interaction data on bone resorption. The present study shows that higher dietary acidity, as determined by NEAP score and *IL6* polymorphism at the -572 CC genotype, may accentuate the rate of bone resorption. Studies on the effects of dietary acidity on bone have yielded mixed and inconsistent findings. While some studies suggested that high dietary acidity are detrimental to bone [39,45,70], other studies reported no association between dietary acidity and the risk of osteoporosis [66,71,72]. With the contrasting results, we tried to reconcile the inconclusive findings of dietary acidity with the interaction of *IL6* SNPs, but to no avail. Although the interaction between *IL6* -572G/C and dietary acidity was not statistically significant in the analysis, the replication of this interaction in another large population study is highly warranted.

An important strength in our study was that it was population-based. Bone health is highly dependent on heritability, and a population-based study may minimize the bias of the result. There are several limitations in this study. Firstly, it was a cross-sectional study, and hence we were unable to determine the causality between the consumption of acid ash diet/*IL6* SNP and CTX1. Future studies should also include the investigation of long-term changes in IL6 and the quantification of bone mineral. Secondly, the acid ash diet was estimated from dietary intake. The absorption of individual ions from food is variable and quite complex. It is highly dependent on an individual’s gastrointestinal absorption of the nutrients from food and also on kidney function to filter the net acid excretion. Thus, measurement bias in determining the acid ash diet’s effect on bone health was one of the major limitations that could not be avoided. In addition, although we have included the confounders in the analysis, other potential confounding factors such as gene–gene interactions due to other potential candidate genes (vitamin D receptor [73], estrogen receptor beta (*ERβ*) [74], transforming growth factor-β1 (*TGF-β1*) [75], C-reactive protein (CRP), adiponectin, and tumor necrosis factor-alpha (TNF-α) [76] were not included in this study due to financial and resource constraints. Last but not least, other biochemical parameters which may influence bone remodeling as suggested in several studies [77,78,79]—for example, estrogen, testosterone, parathyroid hormone (PTH), and blood PH—were not measured in this study, which limits our comprehensive interpretation of the results. Despite the fact that the current study used FFQ as a dietary assessment tool, and this is generally accepted as a better dietary assessment tool for reflecting habitual dietary intake as compared to food records, nevertheless, it is inevitable that food intake measurement is based on self-reported data which have inherent limitations and may limit the finding interpretation of the association between bone turnover markers such as CTX-1 and dietary acid load. Future studies should consider the use of biomarkers capable of objectively assessing food consumption without the bias of self-reported dietary assessment.

## 5. Conclusions

In summary, our study confirms that the *IL6* polymorphism at -174 G/C is rare in the Chinese population and does not contribute significantly to CTX1. Besides this, our results also do not support the interaction effect of dietary acidity and *IL6*-572 G/C with CTX1. These data add to the limited literature regarding the interaction between DAL and the *IL6* polymorphism associated with bone health, especially in Asian populations. It is suggested that future studies should include other bone biomarkers for bone resorption and formation, and should be accompanied by more time points of assessment for the better understanding of the role of genetic polymorphism and dietary acidity in bone health.

## Figures and Tables

**Table 1:** Characteristics of the study participants.

	*n* (%)	Mean ± SD
**Social demographics**		
Age (year)		67 ± 7
Years of menopause (year)		16 ± 8
Educational level (years)		8 ± 5
**Anthropometrics**		
Weight (kg)		57.91 ± 9.6
Height (m)		1.54 ± 0.1
Waist circumference (cm)		80.3 ± 9.1
Body fat percentage (%)		35.1 ± 5.2
BMI (kg/m^2^)		24.3 ± 3.8
Underweight (<18.5)	10 (5.0)	
Normal (18.5–24.9)	113 (55.9)	
Overweight (25.0–29.9)	64 (31.7)	
Obese (≥ 30)	15 (7.4)	
**Lifestyles**		
Physical activity (MET-min/week)		
Below recommendation (<600 MET)	77 (37.9)	
Meeting recommendation (≥600 MET)	126 (62.1)	
NEAP score (mEq/day)		72.8 ± 28.7
Normal (<72.8)	114 (56.2)	
Elevated (≥72.8)	89 (43.8)	
**Biochemical analysis**		
Fasting blood glucose (mmol/L)		5.89 ± 0.93
Serum of 25(OH)D (nmol/L)		
Deficiency (<30)	66 (32.5)	
Inadequate (30–50)	100 (49.3)	
Adequate (>50)	37 (18.2)	
CTX-1 (ng/mL)		0.445 ± 0.198
Normal (<0.445)	116 (57.1)	
Elevated (≥0.445)	87 (42.9)	
**Genetic analysis**		
*IL6* gene -174G/C (genotype)		
CC	0	
CG	0	
GG	203 (100)	
*IL6* gene -572G/C (genotype)		
GG	5 (2.5)	
CG	84 (41.4)	
CC	114 (56.1)	

Data are presented as mean ± SD or frequency (percentage).

**Table 2:** Correlations between selected sociodemographic background, anthropometry parameters, biochemical indices, and NEAP with CTX1 among the participants.

	*r*	*p*
NEAP	0.084	0.24
Age (year)	−0.189	0.01 *
Height (m)	0.133	0.06
Serum of 25(OH)D (nmol/L)	−0.105	0.14
Educational level (year)	0.108	0.13
Fasting blood glucose (mmol/L)	−0.101	0.15
Waist circumference (cm)	−0.098	0.16

* *p* < 0.05.

**Table 3:** Demographic, anthropometrics, lifestyle factors, and biochemical analysis according to *IL6* gene -572G/C.

	IL6 rs1800796			
	GG + CG (*n* = 89)	CC (*n* = 114)	*t*-test	*p*
**Social demographics**				
Age (year)	65.91 ± 5.7	67.17 ± 7.1	−1.36	0.18
Years of menopause (year)	15.47 ± 6.8	16.63 ± 8.4	−1.08	0.28
Marital status				
Single	8 (9)	10 (8.8)		
Married	67 (75.3)	90 (78.9)		
Divorced	2 (2.2)	4 (3.5)		
Others (widow or widower)	12 (13.5)	10 (8.8)		
Educational level (years)	8.54 ± 4.7	7.45 ± 4.5	1.24	0.22
**Anthropometrics**				
Weight (kg)	57.23 ± 9.4	58.44 ± 9.8	−0.883	0.38
Height (m)	1.54 ± 0.1	1.54 ± 0.1	0.155	0.88
Waist circumference (cm)	79.74 ± 8.7	80.72 ± 9.4	−0.753	0.45
Body fat percentage (%)	34.96 ± 5.5	35.26 ± 5.04	−0.400	0.69
BMI (kg/m^2^)	24.1 ± 3.7	24.5 ± 3.9	−0.628	0.53
**Lifestyles factors**				
Physical activity (MET-min/week)				
Below recommendation (<600 MET)	30 (33.7)	47 (41.2)		
Meeting recommendation (≥600 MET)	59 (66.3)	67 (58.8)		
NEAP (mEq/day)	74.97 ± 29.4	71.12 ± 28.2	0.945	0.35
**Biochemical analysis**				
Fasting blood glucose (mmol/L)	5.92 ± 0.95	5.87 ± 0.9	0.397	0.69
Serum of 25(OH) D (nmol/L)				
Deficiency	28 (31.5)	38 (33.3)		
Inadequate	44 (49.4)	56 (49.1)		
Adequate	17 (19.1)	20 (17.5)		
CTX-1 (ng/mL)	0.42 ± 0.19	0.46 ± 0.21	−1.54	0.12

Data are presented as mean ± SD or frequency (percentage); difference between the means of two independent groups was measured by independent samples test.

**Table 4:** Hierarchical linear regression analyses for the association between the selected variables and CTX1.

Variables	Step 1			Step 2			Step 3		
	Beta	*t*	*p*	Beta	*t*	*p*	Beta	*t*	*p*
Age (year)	−0.143	−1.91	0.057	−0.168	−2.26	0.025	−0.166	−2.21	0.028
Height (m)	0.083	1.16	0.25	0.083	1.17	0.24	0.084	1.19	0.24
Serum of 25(OH)D (nmol/L)	−0.108	−1.54	0.13	−0.114	−1.64	0.10	−0.115	−1.64	0.10
Educational level (years)	0.036	0.494	0.62	0.072	0.978	0.33	0.075	1.004	0.32
Fasting blood glucose (mmol/L)	−0.053	−0.728	0.47	−0.058	−0.797	0.43	−0.055	−0.756	0.45
Waist circumference (cm)	−0.077	−1.03	0.30	−0.079	−1.07	0.28	−0.080	−1.09	0.28
NEAP (mEq/day)				0.153	2.18	0.031	0.173	1.66	0.098
*IL6* gene -572G/C (GG + CG = 0, CC = 1)				0.140	2.03	0.044	0.188	0.977	0.33
NEAP x IL6 gene -572G/C							−0.054	−0.266	0.79

Step 1: F (6, 196) = 2.32, *p* = 0.035, R^2^ = 0.066; Step 2: F (8, 194) = 2.81, *p* = 0.006, ΔR^2^ = 0.038, ΔF (2, 194) = 4.07, *p*_(ΔF)_ = 0.018; Step 3: F (9, 193) = 2.49, *p* = 0.010, ΔR^2^ = 0.000, ΔF (1, 193) = 0.071, *p*_(ΔF)_ = 0.79. 2 Step 1 included all the adjusted variables (age, height, serum of 25(OH)D, education level, fasting blood glucose, and waist circumference); Step 2 included the 2 independent variables of interest (NEAP and *IL6*); Step 3 added the interaction term of NEAP*IL6.
